# Supplementary material for: Hybrid Models and Biological Model Reduction with PyDSTool
Source: PLoS Comput Biol. 2012 Aug 9;8(8):e1002628. doi: 10.1371/journal.pcbi.1002628 (PMC3415397; doi:10.1371/journal.pcbi.1002628)
Supplement: Text S4 — Complete source code for the PyDSTool package (version 0.88.120504). Includes API documentation and help files linking to web pages. This file is identical to the current public release on Sourceforge.net. (ZIP) [file pcbi.1002628.s004.zip › PyDSTool/html/PyDSTool.Toolbox.neuro_data.spike_feature-class.html]

xml version="1.0" encoding="ascii"?


PyDSTool.Toolbox.neuro\_data.spike\_feature


| Home | Trees | Indices | Help | PyDSTool |
| --- | --- | --- | --- | --- |

Package PyDSTool ::
Package Toolbox ::
Module neuro\_data ::
Class spike\_feature

[hide private]

[frames] | no frames]

# Class spike\_feature

source code

```
       object --+            
                |            
 MProject.feature --+        
                    |        
MProject.feature_node --+    
                        |    
 MProject.qt_feature_node --+
                            |
                           spike_feature
```

---

pars keys: tol


|  |  |
| --- | --- |
| Instance Methods | [hide private] |

|  |  |
| --- | --- |
| \_local\_init(self) | source code |

|  |  |
| --- | --- |
| evaluate(self, target)  Default method: evaluate sub-features in order (assumes they are stored as a list). | source code |

**Inherited from `MProject.feature_node`**:
`__getitem__`,
`__init__`,
`__repr__`,
`__str__`,
`propagate_verbosity`,
`set_ref_traj`

**Inherited from `MProject.feature_node`** (private):
`_residual_info`

**Inherited from `MProject.feature`**:
`__call__`,
`__eq__`,
`__hash__`,
`__ne__`,
`finish`,
`info`,
`postprocess_ref_traj`,
`prepare`,
`reset_metric`,
`validate`

**Inherited from `MProject.feature`** (private):
`_find_idx`

**Inherited from `object`**:
`__delattr__`,
`__getattribute__`,
`__new__`,
`__reduce__`,
`__reduce_ex__`,
`__setattr__`


|  |  |
| --- | --- |
| Properties | [hide private] |

**Inherited from `object`**:
`__class__`


|  |  |
| --- | --- |
| Method Details | [hide private] |

|  |  |
| --- | --- |
| evaluate(self, target) | source code |

Default method: evaluate sub-features in order (assumes they are
stored as a list).

Can override with more sophisticated method (e.g. for use with a
dictionary of sub-features).

Overrides: MProject.feature.evaluate

  


| Home | Trees | Indices | Help | PyDSTool |
| --- | --- | --- | --- | --- |

|  |  |
| --- | --- |
| Generated by Epydoc 3.0.1 on Fri May 4 15:24:09 2012 | http://epydoc.sourceforge.net |
